# Worldwide Disseminated IncX4 Plasmid Carrying *mcr-1* Arrives to Wild Mammal in Portugal

**DOI:** 10.1128/spectrum.01245-22

**Published:** 2022-11-17

**Authors:** Josman Dantas Palmeira, Mónica V. Cunha, Helena Ferreira, Carlos Fonseca, Rita Tinoco Torres

**Affiliations:** a Department of Biology & CESAM, University of Aveiro, Campus de Santiago, Aveiro, Portugal; b Centre for Ecology, Evolution and Environmental Changes (cE3c), Faculdade de Ciências da Universidade de Lisboa, Lisbon, Portugal; c Biosystems & Integrative Sciences Institute (BioISI), Faculdade de Ciências da Universidade de Lisboa, Lisbon, Portugal; d UCIBIO Applied Molecular Biosciences Unit, REQUIMTE, University of Porto, Porto, Portugal; e Microbiology, Biological Sciences Department, Faculty of Pharmacy, University of Porto, Porto, Portugal; f ForestWISE Collaborative Laboratory for Integrated Forest & Fire Management, Quinta de Prados, Vila Real, Portugal; Instituto de Higiene

**Keywords:** colistin resistance, IncX4, interface human-animal-environment, wild mammals, *mcr-1*

## Abstract

The *mcr-1* gene spread is worldwide recognized as a public health threat at multidrug-resistant infections therapy level. Here, we report for the first time, to the best of our knowledge, the detection of the globally distributed IncX4 plasmid carrying *mcr-1* (*mcr-1*/IncX4) in Escherichia coli isolated from a wild mammal in Portugal and Europe. This plasmid was found in a ST533 E. coli isolate with a multidrug-resistant profile, virulence potential, and possibly phylogenetically related to human isolates. Our work contributes to highlight the importance of antimicrobial resistance (AMR) surveillance in wildlife, an important compartment of the whole ecosystem often overlooked in the fight against AMR.

**IMPORTANCE** Colistin resistance mediated by plasmids is recognized worldwide as an emergency problem connected with the whole ecosystem, since is well described in the interface of the human-animal-environment. The plasmid IncX4 is reported as one of the most prevalent plasmids harboring the gene *mcr-1*. On an European scale the plasmid IncX4 carrying *mcr-1* has been described in humans, the environment, and animals, including wildlife, but only in wild birds. This study shows the first report of the plasmid IncX4 harboring *mcr-1* in a wild mammal in Portugal and Europe, identified in a ST533 E. coli commensal that is, curiously, more related to isolates from humans than from livestock. Our findings show that the plasmid IncX4 harboring *mcr-1* is well established in a colistin resistance drive embracing the whole ecosystem.

## OBSERVATION

Since the first report of the *mcr-1* gene, plasmid-mediated colistin resistance has been described worldwide in a variety of *Enterobacterales* from different origins (e.g., human, livestock, wildlife, and environmental), highlighting the need for the operationalization of the One Heath framework (1). Today, the description of bacteria harboring *mcr-1* genes is global, with broad ecosystem coverage, from humans to a variety of wildlife species (1), stressing its global dissemination. This worldwide dissemination is mainly attributable to the successful horizontal transfer of *mcr-1* carrying plasmids, such as IncX4 (1–3). Here, we report for the first time, to the best of our knowledge, the detection of the globally distributed IncX4 plasmid carrying *mcr-1* (*mcr-1*/IncX4) in Escherichia coli isolated from a wild ungulate in Europe. We also show through the whole-genome sequence (WGS) that this strain is related to isolates from humans, harboring high pathogenic potential and a genetic pool of antimicrobial resistance determinants.

The *mcr-1*/IncX4-positive E. coli strain U147 was isolated from a fecal sample opportunistically collected from an adult fallow deer (Dama dama) female hunted in the south of Portugal (Évora district, −7,667904, 38,224423). The animal was hanted under the hunting season 2019/2020 and the sample collect in January of 2020. The hunting of wild deer in Portugal occurs under cultural/traditional context, and the animals’ meat is used for human consumption. The U147 isolate was recovered in the frame of a national surveillance project assessing antimicrobial resistance (AMR) in wild ungulates microbiota. This isolate was selected after preliminary enrichment of the fecal sample in Tryptic Soy Broth (TSB, Liofilchem, Italy), inoculation onto MacConkey agar (Liofilchem, Italy) supplemented with ampicillin (100 μg/mL, Sigma-Aldrich, Germany) (4). API20E (bioMérieux, France) testing identified the selected isolate as presumptive E. coli. Under EUCAST standard guidelines, antimicrobial susceptibility was determined by agar disk-diffusion (Oxoid, United Kingdom), and the MIC for colistin (0,125μg-256/mL) was determined by the broth microdilution assay. The U147 isolate exhibited resistance to ampicillin, ciprofloxacin, gentamicin, tetracycline, sulfamethoxazole+trimethoprim and chloramphenicol, indicating phenotypic multidrug resistance (MDR) (5). The colistin MIC was 2 μg/mL which, according to ECOFF criteria, indicates a wild-type phenotype.

The mcr genes were screened by PCR, and later confirmed by WGS. Genomic DNA extraction, library preparation, sequencing, and read trimming was performed by MicrobesNG (https://microbesng.uk/). Genomic DNA libraries were prepared following the manufacturer’s indications, using the Nextera XT Library Preparation kit (Illumina, San Diego, USA), and sequenced by Illumina (San Diego, CA, USA) using a 250-bp paired-end protocol. *De novo* assembly was performed with Unicycler v0.5.0 (6). The assembly graph was visualized with Bandage v0.8.1 (7). We then used QUAST web interface to check the quality of the assembly (8). Genome-based taxonomy was performed in the Type (Strain) Genome Server (9). The phylogenetic group was determined by a PCR protocol (10) and confirmed by ClermonTyping-tool (http://clermontyping.iame-research.center/) (11). The MLST profile was assessed with the MLST-2.0 server (https://cge.food.dtu.dk/services/MLST/) according to Achtman's MLST scheme (12). The core genome MLST was evaluated with cgMLSTFinder-1.1 (https://cge.food.dtu.dk/services/cgMLSTFinder/). We used SerotypeFinder-2.0.1 (https://cge.food.dtu.dk/services/SerotypeFinder/) to identify the serotype and CHTyper-1.0 (https://cge.food.dtu.dk/services/CHTyper/) to predict the FimH type and FumC type. Plasmids’ incompatibility groups were assessed by the PlasmidFinder database (nucleotide sequences downloaded on the 8th June 2022) available from abricate v1.0.1 (https://github.com/tseemann/abricate). We used MOB-suite v3.1.0 to predict the relaxase type, mate-pair formation type and predicted transferability of the *mcr*-carrying plasmid (13). To ascertain the AMR genotype, we used the ResFinder database (nucleotide sequences downloaded on 7 June 2022) from abricate v1.0.1 and RGI-5.2.0 (https://card.mcmaster.ca/analyze/rgi).

A total of 1,440,780 paired-end trimmed reads with a mean of 136 coverage were generated. The reads were assembled into 103 contigs, with a total length of 5037902-bp and a GC content of 50.77%. Six extrachromosomal elements were fully resolved and circularized (Fig. S1).

**FIG 1:**
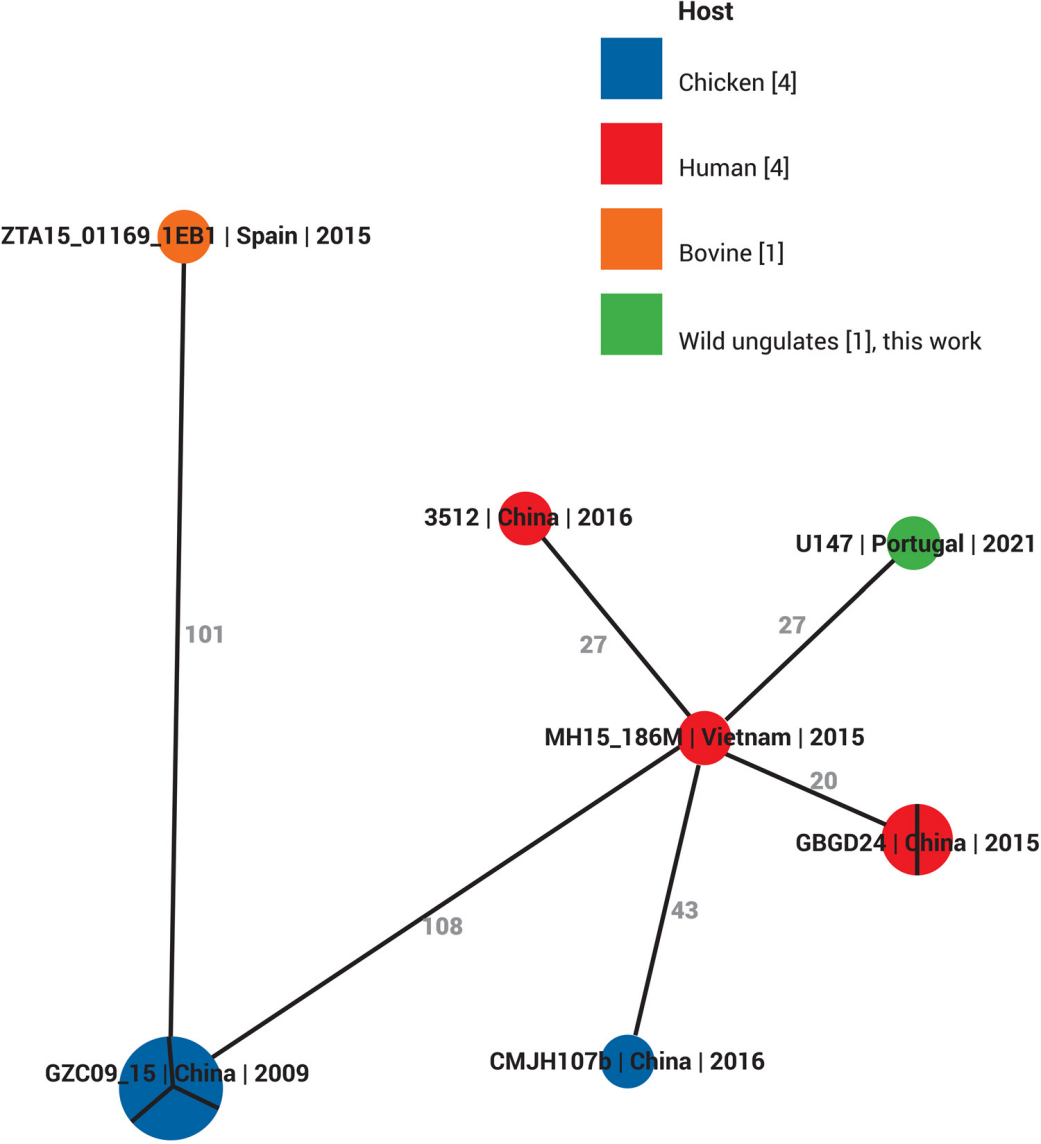
Phylogenetic tree of the 10 publicly available cgST138832 Escherichia coli sequences based on core genome MLST with 200 allelic differences as maximum threshold, using the BacWGSTdb-webtool (http://bacdb.cn/BacWGSTdb/analysis_single.php).

We found that the U147 strain belongs to the E. coli species and to the phylogenetic group B1. The serotype of E. coli U147 was determined as O9/O9a:H10 and CH-Type 4–31. The U147 E. coli strain belongs to the sequence type (ST) 533 and to the core genome (cg) ST138832. The ST533 E. coli have been reported in Europe and Asia, in humans, animals (livestock, pets and wild birds) and the wider environment. In all descriptions, the isolates harbored AMR determinants (14, 15). Previously reported studies support that this ST can be involved in the E. coli, without and with AMR determinant, transmission between humans and animals (16). Phylogenetic analysis (Fig. 1) suggests that the U147 E. coli can be more related to human origin isolates than livestock. It is difficult to stablish the origin of this *mcr-1/*IncX4 plasmid, but draws attention that this fallow deer shared the habitat with another wild animals, such as wild boar and red deer, being all these animals have contact with large range areas, due to their daily movement features, and inhabited a touristic fenced hunting ground on southeast of central Portugal, what create the opportunity for animal be exposure to anthropogenic pressure since that the turnover of different people in this area is very high.

We found 95 virulence determinants in U147 strain, all located in the predicted chromosomal contigs (Table S1). Furthermore, we observed were along with the presence of 697 proteins assigned to pathogenic families (https://cge.food.dtu.dk/services/PathogenFinder/), highlighting the high pathogenic potential of this isolate. We also found multiple AMR genes, with the majority of these genes located in chromosomal contigs with high sequencing depth and linked together in the assembly graph (Table S2 and Fig. S1), suggesting that these genes are colocated in the same chromosomally integrated mobile genetic element (Fig. S2). These genes encode resistance to clinically relevant antibiotic classes, such as beta-lactams, aminoglycosides and tetracyclines. The only exception is the mcr1.1 gene encoding resistance to colistin, which was found in a 33-kb IncX-4 plasmid. Beyond mcr-1.1, the resistance genes present in the U147 includes *bla*_TEM-1b_, *aadA1*, *aadA2*, *aadA17*, *aph(3′)-Ia*, *aac(3)-IId*, *tetA*, *tetM*, *sul2*, *sul3*, *dfrA12*, *cmlA1,* and *floR*.

Of the six extrachromosomal elements, we found plasmid incompatibility groups for five of them, including the *mcr1.1*-carrying IncX4 plasmid (Fig. S1). This multicopy plasmid is 33,304-bp long and is predicted to be self-conjugative (Table S3). The plasmid belongs to the mating-pair formation T (MPFT) family, carries a MOBP relaxase.

The *mcr-1* gene has already been described in wildlife, namely, wild birds in Europe, Asia and South America, and wild mammals in Europe, Africa and Asia (17) but associated with other plasmids. In Portugal, bacteria harboring the *mcr-1*/IncX4 combination have been described in human and livestock samples (18, 19). At the European scale, the *mcr-1*/IncX4 combination has been reported in the human-animal-environment interface, including wild birds in Spain, such as the white stork (20, 21) but, as far as we are aware of, no description in wild mammals has been reported. Our description of the *mcr-1*/IncX4 combination in a wild mammal from Portugal and Europe has great significance because (i) the IncX4 plasmids are known to play a key role on the spread of *mcr-1* whether in a specific environmental context (3) or at the global level (1, 2); and (ii) wild mammals, particularly wild ungulates, are well distributed in Europe and across a broad range of environmental settings, namely, humanized areas, thereafter playing a potential role as drivers of AMR (22, 23).

We report an E. coli strain displaying the *mcr-1*/IncX4 combination in a wild ungulate from Portugal, so far only described in human and livestock samples. As far as we know, this is also the first description of the *mcr-1*/IncX4 combination in a wild mammal in Portugal and in Europe. Due to increased abundance, distribution, and overlap with human settings, wild ungulates carrying AMR determinants in bacteria potentially pathogenic to humans may pose important public health challenges. Furthermore, the need to unravel AMR transmission routes from a top down and bottom-up approach, highlights the importance of our work, which contributes to underline the role of AMR surveillance in wildlife, an important compartment of the whole ecosystem often overlooked in the fight against AMR.

### Ethical statement.

None of the authors were responsible, or involved, in animals’ death, and for this study no animal was sacrificed. All institutional, national, and international guidelines for the use and care of all animals have been followed.

### Data availability.

This Whole Genome Sequencing project has been deposited at DDBJ/EMBL/GenBank under the accession no. JAJNCE000000000.
