# Supplementary material for: Worldwide Disseminated IncX4 Plasmid Carrying mcr-1 Arrives to Wild Mammal in Portugal
Source: Microbiol Spectr. 2022 Nov 17;10(6):e01245-22. doi: 10.1128/spectrum.01245-22 (PMC9769835; doi:10.1128/spectrum.01245-22)
Supplement: Supplemental file 1 — Fig. S1 and S2 and Tables S1 to S3. Download spectrum.01245-22-s0001.pdf, PDF file, 0.4 MB [file spectrum.01245-22-s0001.pdf]

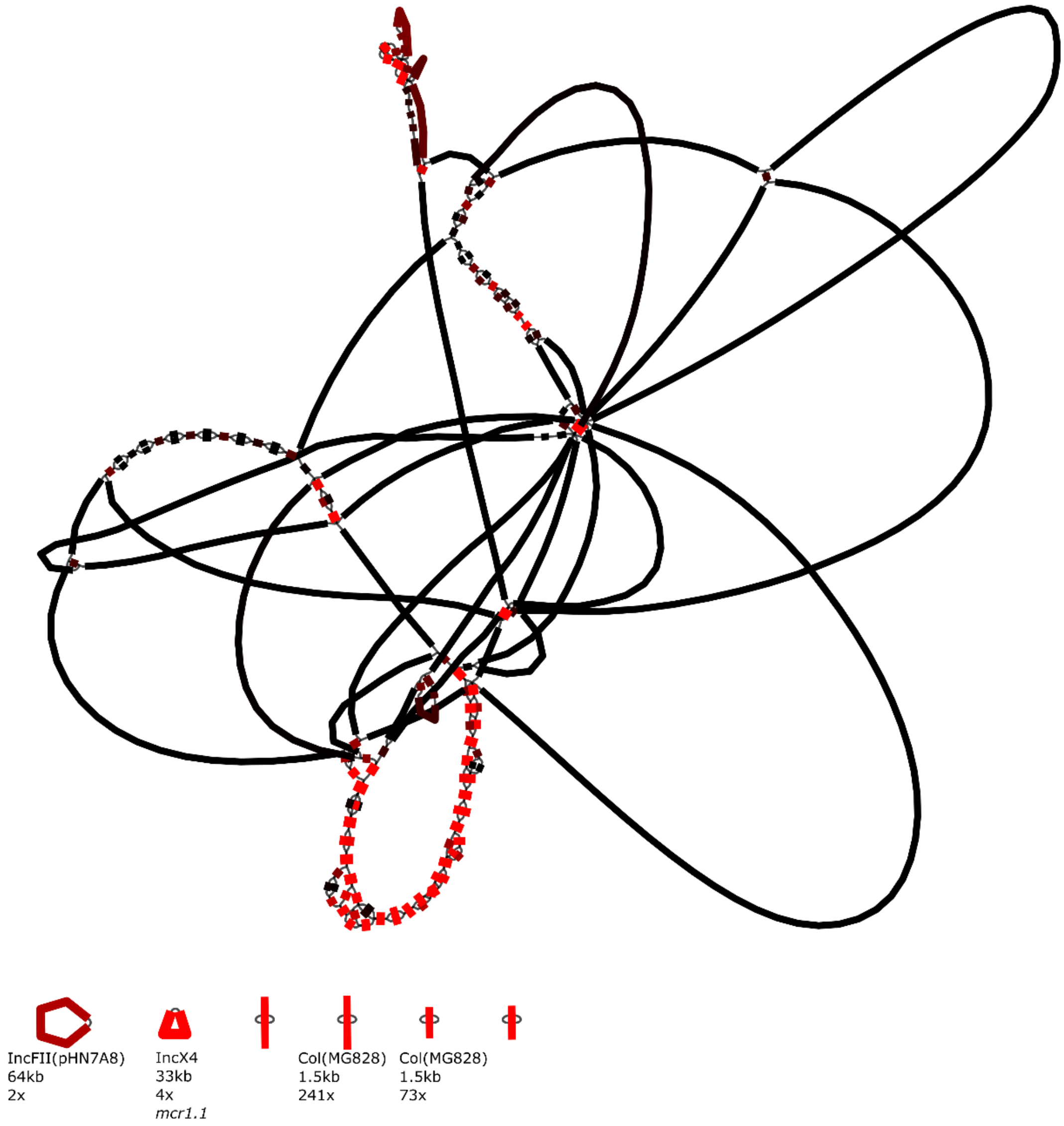

Figure S1. Assembly graph of the whole genome sequencing of U147 *E. coli*, the image was visualized with Bandage v0.8.1.

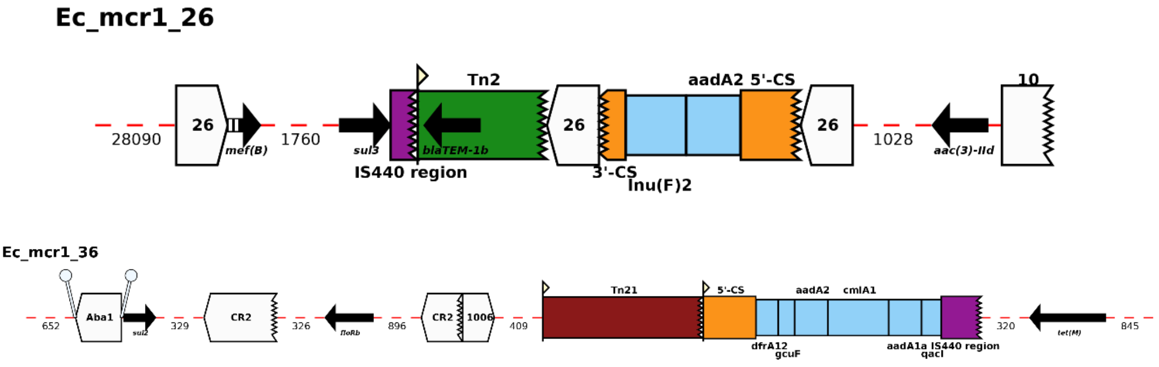

Figure S2. Antimicrobial resistance genes co-located in the same mobile genetic element.

Table S1. Virulence determinants characterization of *E. coli* U147 strain.

CONTIG	START	END	STRAND	GENE	COVERAGE	% COVERAGE	% IDENTITY	ACCESSION	PRODUCT
1	172203	172919	+	*cfaA*	1-717/717	100.00	99.72	WP_000225871	(*cfaA*) colonisation factor antigen a chaperone [Adhesive fimbriae (VF0213) - Adherence (VFC0001)] [*Escherichia coli* E24377A]
1	172949	173449	+	*cfaB*	1-501/501	100.00	100.00	WP_000755097	(*cfaB*) colonization factor antigen 1 [Adhesive fimbriae (VF0213) - Adherence (VFC0001)] [*Escherichia coli* E24377A]
1	173541	176225	+	*cfaC*	1-2685/2685	100.00	99.89	WP_000358674	(*cfaC*) colonisation factor antigen c usher [Adhesive fimbriae (VF0213) - Adherence (VFC0001)] [*Escherichia coli* E24377A]
1	176222	177310	+	*cfaD/cfaE*	1-1089/1089	100.00	100.00	WP_001245988	(*cfaD/cfaE*) minor pilin and initiator [Adhesive fimbriae (VF0213) - Adherence (VFC0001)] [*Escherichia coli* E24377A]
1	313735	314694	+	*gspC*	1-960/960	100.00	95.10	WP_000135089	(*gspC*) general secretion pathway protein C [T2SS (VF0333) - Effector delivery system (VFC0086)] [*Shigella dysenteriae* Sd197]
1	314724	316784	+	*gspD*	1-2046/2046	100.00	97.72	WP_000498818	(*gspD*) general secretion pathway protein D [T2SS (VF0333) - Effector delivery system (VFC0086)] [*Shigella dysenteriae* Sd197]
1	316784	318277	+	*gspE*	1-1494/1494	100.00	96.45	WP_000249362	(*gspE*) general secretion pathway protein E [T2SS (VF0333) - Effector delivery system (VFC0086)] [*Shigella dysenteriae* Sd197]
1	318277	319476	+	*gspF*	1-1200/1200	100.00	96.33	WP_001173470	(*gspF*) general secretion pathway protein F [T2SS (VF0333) - Effector delivery system (VFC0086)] [*Shigella dysenteriae* Sd197]
1	319517	319972	+	*gspG*	1-456/456	100.00	95.39	WP_001087302	(*gspG*) general secretion pathway protein G [T2SS (VF0333) - Effector delivery system (VFC0086)] [*Shigella dysenteriae* Sd197]
1	319976	320539	+	*gspH*	1-564/564	100.00	95.75	WP_001115126	(*gspH*) general secretion pathway protein H [T2SS (VF0333) - Effector delivery system (VFC0086)] [*Shigella dysenteriae* Sd197]
1	320536	320907	+	*gspI*	1-372/372	100.00	94.62	WP_000820136	(*gspI*) general secretion pathway protein I [T2SS (VF0333) - Effector delivery system (VFC0086)] [*Shigella* dysenteriae Sd197]
1	320904	321509	+	*gspJ*	1-606/606	100.00	97.03	WP_001250462	(*gspJ*) general secretion pathway protein J [T2SS (VF0333) - Effector delivery system (VFC0086)] [*Shigella* dysenteriae Sd197]
1	321506	322483	+	*gspK*	1-978/978	100.00	98.47	WP_000633233	(*gspK*) general secretion pathway protein K [T2SS (VF0333) - Effector delivery system (VFC0086)] [*Shigella dysenteriae* Sd197]
1	322480	323658	+	*gspL*	1-1179/1179	100.00	96.18	WP_000097229	(*gspL*) general secretion pathway protein L [T2SS (VF0333) - Effector delivery system (VFC0086)] [*Shigella dysenteriae* Sd197]
1	323660	324196	+	*gspM*	1-537/537	100.00	94.60		(*gspM*) general secretion pathway protein M [T2SS (VF0333) - Effector delivery system (VFC0086)] [*Shigella dysenteriae* Sd197]
11	62722	64014	+	*espX5*	1-1293/1293	100.00	96.06	WP_001270087	(*espX5*) Type III secretion system effector EspX5 [TTSS secreted effectors (VF1111) - Effector delivery system (VFC0086)] [*Escherichia coli* O55:H7 str. CB9615]

11	94950	96530	-	*espX4*	1-1581/1581	99.94	93.11	WP_000900547	(*espX4*) Type III secretion system effector EspX4 [TTSS secreted effectors (VF1110) - Effector delivery system (VFC0086)] [*Escherichia coli* O157:H7 str. EDL933]
13	50258	53405	-	*acrB*	1-3145/3147	99.84	83.81	WP_002892069	(*acrB*) acriflavine resistance protein B [AcrAB (VF0568) - Antimicrobial activity/Competitive advantage (VFC0325)] [*Klebsiella pneumoniae subsp. pneumoniae* NTUH-K2044]
13	101446	102807	+	*allB*	1-1362/1362	99.78	83.08	WP_012737535	(*allB*) allantoinase [Allantion utilization (VF0572) - Nutritional/Metabolic factor (VFC0272)] [*Klebsiella pneumoniae subsp. pneumoniae* NTUH-K2044]
14	77	697	-	*entD*	1-621/621	100.00	93.40	WP_001375021	(*entD*) phosphopantetheinyl transferase component of enterobactin synthase multienzyme complex [Enterobactin (VF0228) - Nutritional/Metabolic factor (VFC0272)] [*Escherichia coli* CFT073]
14	872	3112	-	*fepA*	1-2241/2241	100.00	96.88	WP_001034892	(*fepA*) ferrienterobactin outer membrane transporter [Enterobactin (VF0228) - Nutritional/Metabolic factor (VFC0272)] [*Escherichia coli* CFT073]
14	3355	4557	+	*fes*	1-1203/1203	100.00	96.76	WP_000125846	(*fes*) enterobactin/ferric enterobactin esterase [Enterobactin (VF0228) - Nutritional/Metabolic factor (VFC0272)] [*Escherichia coli* CFT073]
14	4775	8628	+	*entF*	1-3854/3882	99.28	95.54	WP_000077767	(*entF*) enterobactin synthase multienzyme complex component ATP-dependent [Enterobactin (VF0228) - Nutritional/Metabolic factor (VFC0272)] [*Escherichia coli* CFT073]
14	8872	10005	+	*fepE*	1-1134/1134	100.00	91.62	WP_000096764	(*fepE*) LPS O-antigen length regulator [Enterobactin (VF0228) - Nutritional/Metabolic factor (VFC0272)] [*Escherichia coli* CFT073]
14	10002	10817	-	*fepC*	1-816/816	100.00	97.06	WP_000140634	(*fepC*) ferrienterobactin ABC transporter ATPase [Enterobactin (VF0228) - Nutritional/Metabolic factor (VFC0272)] [*Escherichia coli* CFT073]
14	10814	11806	-	*fepG*	1-993/993	100.00	94.46	WP_000640938	(*fepG*) iron-enterobactin ABC transporter permease [Enterobactin (VF0228) - Nutritional/Metabolic factor (VFC0272)] [*Escherichia coli* CFT073]
14	11803	12807	-	*fepD*	1-1005/1005	100.00	95.72	WP_001443194	(*fepD*) ferrienterobactin ABC transporter permease [Enterobactin (VF0228) - Nutritional/Metabolic factor (VFC0272)] [*Escherichia coli* CFT073]
14	12918	14168	+	*entS*	1-1251/1251	100.00	95.60	WP_001041793	(*entS*) enterobactin exporter iron-regulated [Enterobactin (VF0228) - Nutritional/Metabolic factor (VFC0272)] [*Escherichia coli* CFT073]
14	14172	15128	-	*fepB*	1-957/957	100.00	97.49	WP_001234311	(*fepB*) ferrienterobactin ABC transporter periplasmic binding protein [Enterobactin (VF0228) - Nutritional/Metabolic factor (VFC0272)] [*Escherichia coli* CFT073]
14	15503	16678	+	*entC*	1-1176/1176	100.00	97.53	WP_001336900	(*entC*) isochorismate synthase 1 [Enterobactin (VF0228) - Nutritional/Metabolic factor (VFC0272)] [*Escherichia coli* CFT073]

14	16688	18298	+	*entE*	1-1611/1611	100.00	95.59	WP_000026784	(*entE*) 23-dihydroxybenzoate-AMP ligase component of enterobactin synthase multienzyme complex [Enterobactin (VF0228) - Nutritional/Metabolic factor (VFC0272)] [*Escherichia coli* CFT073]
14	18312	19169	+	*entB*	1-858/858	100.00	97.79	WP_001007140	(*entB*) isochorismatase [Enterobactin (VF0228) - Nutritional/Metabolic factor (VFC0272)] [*Escherichia coli* CFT073]
14	19169	19915	+	*entA*	1-747/747	100.00	95.98	WP_000348393	(*entA*) 23-dihydro-23-dihydroxybenzoate dehydrogenase EntA [Enterobactin (VF0228) - Nutritional/Metabolic factor (VFC0272)] [*Escherichia coli* CFT073]
14	101391	101827	-	*fur*	1-437/453	96.47	87.64	NP_459678	(*fur*) ferric iron uptake transcriptional regulator [Fur (VF0113) - Regulation (VFC0301)] [*Salmonella enterica subsp. enterica* serovar Typhimurium str. LT2]
16	89124	90115	-	*rpoS*	2-993/993	99.80	92.85	NP_461845	(*rpoS*) RNA polymerase sigma factor RpoS [RpoS (VF0112) - Regulation (VFC0301)] [*Salmonella enterica subsp. enterica* serovar Typhimurium str. LT2]
17	49067	49775	-	*yagV/ecpE*	1-709/711	99.72	97.04	WP_001301550	(*yagV/ecpE*) E. coli common pilus chaperone EcpE [ECP (VF0404) - Adherence (VFC0001)] [*Escherichia coli* O157:H7 str. EDL933]
17	49744	51387	-	*yagW/ecpD*	1-1644/1644	100.00	98.30	WP_001265657	(*yagW/ecpD*) polymerized tip adhesin of ECP fibers [ECP (VF0404) - Adherence (VFC0001)] [*Escherichia coli* O157:H7 str. EDL933]
17	51377	53902	-	*yagX/ecpC*	1-2526/2526	100.00	98.77	WP_001131063	(*yagX/ecpC*) E. coli common pilus usher EcpC [ECP (VF0404) - Adherence (VFC0001)] [*Escherichia coli* O157:H7 str. EDL933]
17	53928	54596	-	*yagY/ecpB*	1-669/669	100.00	98.66	WP_000716386	(*yagY/ecpB*) E. coli common pilus chaperone EcpB [ECP (VF0404) - Adherence (VFC0001)] [*Escherichia coli* O157:H7 str. EDL933]
17	54654	55241	-	*yagZ/ecpA*	1-588/588	100.00	99.32	WP_000730972	(*yagZ/ecpA*) E. coli common pilus structural subunit EcpA [ECP (VF0404) - Adherence (VFC0001)] [*Escherichia coli* O157:H7 str. EDL933]
17	55316	55858	-	*ykgK/ecpR*	1-543/543	100.00	97.79	WP_000389022	(*ykgK/ecpR*) regulator protein EcpR [ECP (VF0404) - Adherence (VFC0001)] [*Escherichia coli* O157:H7 str. EDL933]
17	58926	63167	+	*fdeC*	1-4245/4251	99.76	92.58	WP_000092542	(*fdeC*) adhesin FdeC [FdeC (VF0506) - Adherence (VFC0001)] [*Escherichia coli* O45:K1:H7 str. S88]
2	191142	191792	-	*rcsB*	1-651/651	100.00	84.64	WP_002913007	(*rcsB*) transcriptional regulator RcsB [RcsAB (VF0571) - Regulation (VFC0301)] [*Klebsiella pneumoniae subsp. pneumoniae* NTUH-K2044]
2	369634	370524	+	*galF*	1-891/891	100.00	94.16	WP_001741945	(*galF*) GalU regulator GalF [Capsule (VF0560) - Immune modulation (VFC0258)] [*Klebsiella pneumoniae subsp. pneumoniae* NTUH-K2044]
2	370918	371546	+	KP1_RS17355	1-629/630	99.84	91.57	WP_014907219	(KP1_RS17355) phosphatase PAP2 family protein [Capsule (VF0560) - Immune modulation (VFC0258)] [*Klebsiella pneumoniae subsp. pneumoniae* NTUH-K2044]

2	372508	373941	+	KP1_RS17345	1-1434/1434	100.00	88.01	WP_041937790	(KP1_RS17345) capsule assembly Wzi family protein [Capsule (VF0560) - Immune modulation (VFC0258)] [*Klebsiella pneumoniae subsp. pneumoniae* NTUH-K2044]
2	374086	375216	+	KP1_RS17340	1-1131/1134	99.74	80.11	WP_014907221	(KP1_RS17340) polysaccharide export protein [Capsule (VF0560) - Immune modulation (VFC0258)] [*Klebsiella pneumoniae subsp. pneumoniae* NTUH-K2044]
2	385747	387148	+	*gndA*	1-1402/1407	99.64	93.30	WP_014907233	(*gndA*) NADP-dependent phosphogluconate dehydrogenase [Capsule (VF0560) - Immune modulation (VFC0258)] [*Klebsiella pneumoniae subsp. pneumoniae* NTUH-K2044]
2	387344	388510	+	*ugd*	1-1167/1167	100.00	96.31	WP_004175261	(*ugd*) UDP-glucose 6-dehydrogenase [Capsule (VF0560) - Immune modulation (VFC0258)] [*Klebsiella pneumoniae subsp. pneumoniae* NTUH-K2044]
2	390377	391792	+	KP1_RS17280	1-1416/1416	100.00	94.21	WP_014907234	(KP1_RS17280) mannose-1-phosphate guanylyltransferase/mannose-6-phosphate isomerase [Capsule (VF0560) - Immune modulation (VFC0258)] [*Klebsiella pneumoniae subsp. pneumoniae* NTUH-K2044]
2	391816	393158	+	*rfbK1*	1-1343/1371	97.96	95.53	WP_004899416	(*rfbK1*) O9 family phosphomannomutase RfbK1 [Capsule (VF0560) - Immune modulation (VFC0258)] [*Klebsiella pneumoniae subsp. pneumoniae* NTUH-K2044]
2	467232	469253	-	*fyuA/psn*	1-2022/2022	100.00	99.90	WP_002212885	(*fyuA/psn*) pesticin/yersiniabactin receptor protein [Yersiniabactin (VF0136) - Nutritional/Metabolic factor (VFC0272)] [*Yersinia pestis* CO92]
2	469384	470961	-	*ybtE*	1-1578/1578	100.00	99.87	WP_001088826	(*ybtE*) yersiniabactin siderophore biosynthetic protein [Yersiniabactin (VF0136) - Nutritional/Metabolic factor (VFC0272)] [*Yersinia pestis* CO92]
2	470965	471768	-	*ybtT*	1-804/804	100.00	99.88	WP_002212799	(*ybtT*) type II thioesterase YbtT [Yersiniabactin (VF0136) - Nutritional/Metabolic factor (VFC0272)] [*Yersinia pestis* CO92]
2	471765	472865	-	*ybtU*	1-1101/1101	100.00	99.91	WP_000982866	(*ybtU*) yersiniabactin biosynthetic protein YbtU [Yersiniabactin (VF0136) - Nutritional/Metabolic factor (VFC0272)] [*Yersinia pestis* CO92]
2	472862	482353	-	*irp1*	1-9492/9492	100.00	99.74	WP_002212777	(*irp1*) yersiniabactin biosynthetic protein Irp1 [Yersiniabactin (VF0136) - Nutritional/Metabolic factor (VFC0272)] [*Yersinia pestis* CO92]
2	482441	488548	-	*irp2*	1-6108/6108	100.00	99.72	WP_002212775	(*irp2*) yersiniabactin biosynthetic protein Irp2 [Yersiniabactin (VF0136) - Nutritional/Metabolic factor (VFC0272)] [*Yersinia pestis* CO92]
2	488739	489698	-	*ybtA*	1-960/960	100.00	99.38	WP_000140406	(*ybtA*) transcriptional regulator YbtA [Yersiniabactin (VF0136) - Nutritional/Metabolic factor (VFC0272)] [*Yersinia pestis* CO92]
2	489955	491667	+	*ybtP*	1-1713/1713	100.00	99.53	WP_001327262	(*ybtP*) yersiniabactin ABC transporter ATP-binding/permease protein YbtP [Ybt (VF0564) - Nutritional/Metabolic factor (VFC0272)] [*Klebsiella pneumoniae subsp. pneumoniae* NTUH-K2044]

2	491654	493456	+	*ybtQ*	1-1803/1803	100.00	99.78	WP_002212761	(*ybtQ*) yersiniabactin ABC transporter ATP-binding/permease protein YbtQ [Yersiniabactin (VF0136) - Nutritional/Metabolic factor (VFC0272)] [*Yersinia pestis* CO92]
2	493449	494729	+	*ybtX*	1-1281/1281	100.00	99.38	WP_001286280	(*ybtX*) putative signal transducer [Yersiniabactin (VF0136) - Nutritional/Metabolic factor (VFC0272)] [*Yersinia pestis* CO92]
2	494757	496061	+	*ybtS*	1-1305/1305	100.00	99.69	WP_000703040	(*ybtS*) salicylate synthase Irp9 [Yersiniabactin (VF0136) - Nutritional/Metabolic factor (VFC0272)] [*Yersinia pestis* CO92]
24	18965	20698	+	*ibeC*	1-1734/1734	100.00	97.06	WP_000556298	(*ibeC*) phosphoethanolamine transferase CptA [Ibes (VF0237) - Invasion (VFC0083)] [*Escherichia coli* O45:K1:H7 str. S88]
28	24866	25921	+	*espR1*	198-1253/1260	83.81	94.41	WP_000671691	(*espR1*) Type III secretion system effector espR1 [TTSS secreted effectors (VF1111) - Effector delivery system (VFC0086)] [*Escherichia coli* O55:H7 str. CB9615]
3	118694	119746	-	*ompA*	1-1041/1041	100.00	94.30	AAF37887	(*ompA*) outer membrane protein A [OmpA (VF0236) - Invasion (VFC0083)] [*Escherichia coli* O18:K1:H7 str. RS218]
3	190698	191531	-	*cgsG*	1-834/834	100.00	97.60	WP_001189322	(*cgsG*) curli production assembly/transport protein CsgG [Curli fibers (VF1138) - Adherence (VFC0001)] [*Escherichia coli* O25b:H4-ST131]
3	191558	191974	-	*cgsF*	1-417/417	100.00	98.56	WP_001264082	(*cgsF*) curli production assembly/transport protein CsgF [Curli fibers (VF1138) - Adherence (VFC0001)] [*Escherichia coli* O25b:H4-ST131]
3	191999	192388	-	*cgsE*	1-390/390	100.00	98.97	WP_000833288	(*cgsE*) curli production assembly/transport protein CsgE [Curli fibers (VF1138) - Adherence (VFC0001)] [*Escherichia coli* O25b:H4-ST131]
3	192393	193043	-	*cgsD*	1-651/651	100.00	99.08	WP_000481500	(*cgsD*) transcriptional regulator CsgD [Curli fibers (VF1138) - Adherence (VFC0001)] [*Escherichia coli* O25b:H4-ST131]
3	194574	195029	+	*csgB*	1-456/456	100.00	99.56	WP_000791650	(*csgB*) curlin minor subunit CsgB [Curli fibers (VF1138) - Adherence (VFC0001)] [*Escherichia coli* O25b:H4-ST131]
3	195070	195525	+	*csgA*	1-459/459	99.35	91.50	WP_000771429	(*csgA*) curlin major subunit CsgA [Curli fibers (VF1138) - Adherence (VFC0001)] [*Escherichia coli* O25b:H4-ST131]
3	195584	195916	+	*csgC*	1-333/333	100.00	98.50	WP_000992818	(*csgC*) curli assembly protein CsgC [Curli fibers (VF1138) - Adherence (VFC0001)] [*Escherichia coli* O25b:H4-ST131]
3	280504	281173	-	*phoP*	4-673/675	99.26	81.94	NP_460201	(*phoP*) response regulator in two-component regulatory system with PhoQ [PhoPQ (VF0111) - Regulation (VFC0301)] [*Salmonella enterica subsp. enterica* serovar Typhimurium str. LT2]
30	6645	7625	-	STM0267	1-981/996	98.49	82.57	NP_459264	(STM0267) sciB domain protein [SCI (Salmonella centrisome island) (VF0974) - Effector delivery system (VFC0086)] [*Salmonella enterica subsp. enterica* serovar Typhimurium str. LT2]
30	7622	9505	-	STM0268	1-1884/1884	99.95	84.03	NP_459265	(STM0268) type VI secretion protein [SCI (Salmonella centrisome island) (VF0974) - Effector delivery system (VFC0086)] [*Salmonella enterica subsp. enterica* serovar Typhimurium str. LT2]

30	15640	16140	+	STM0273	1-501/543	92.27	86.23	NP_459270	(STM0273) type VI secretion system-associated protein [SCI (Salmonella centrisome island) (VF0974) - Effector delivery system (VFC0086)] [*Salmonella enterica subsp. enterica* serovar Typhimurium str. LT2]
30	16278	17724	+	STM0274	63-1509/1509	95.89	83.21	NP_459271	(STM0274) EvpB family type VI secretion protein [SCI (Salmonella centrisome island) (VF0974) - Effector delivery system (VFC0086)] [*Salmonella enterica subsp. enterica* serovar Typhimurium str. LT2]
30	17783	18262	+	*tae4*	7-486/486	98.77	80.42	NP_459275	(*tae4*) hypothetical protein [SCI (Salmonella centrisome island) (VF0974) - Effector delivery system (VFC0086)] [*Salmonella enterica subsp. enterica* serovar Typhimurium str. LT2]
30	18744	19229	+	STM0279	1-486/486	100.00	88.68	NP_459277	(STM0279) Hcp1 family type VI secretion system effector [SCI (Salmonella centrisome island) (VF0974) - Effector delivery system (VFC0086)] [*Salmonella enterica subsp. enterica* serovar Typhimurium str. LT2]
30	19834	21177	+	STM0281	1-1344/1344	100.00	80.58	NP_459279	(STM0281) type VI secretion system-associated protein [SCI (Salmonella centrisome island) (VF0974) - Effector delivery system (VFC0086)] [*Salmonella enterica subsp. enterica* serovar Typhimurium str. LT2]
30	23466	27330	+	STM0285	1-3868/3870	99.56	82.22	NP_459283	(STM0285) hypothetical protein [SCI (Salmonella centrisome island) (VF0974) - Effector delivery system (VFC0086)] [*Salmonella enterica subsp. enterica* serovar Typhimurium str. LT2]
30	29284	31184	+	STM0289	1-1886/2190	85.62	82.64	NP_459287	(STM0289) type VI secretion protein Vgr [SCI (Salmonella centrisome island) (VF0974) - Effector delivery system (VFC0086)] [*Salmonella enterica subsp. enterica* serovar Typhimurium str. LT2]
35	10830	12212	-	*ibeB*	1-1383/1383	100.00	96.75	WP_000074207	(*ibeB*) Cu(+)/Ag(+) efflux RND transporter outer membrane channel CusC [Ibes (VF0237) - Invasion (VFC0083)] [*Escherichia coli* O45:K1:H7 str. S88]
4	220528	222426	+	*espL1*	1-1898/1898	100.00	98.53		(*espL1*) Type III secretion system effector espL1 [TTSS secreted effectors (VF1111) - Effector delivery system (VFC0086)] [*Escherichia coli* O55:H7 str. CB9615]
5	96636	97538	-	*fimH*	1-903/903	100.00	97.78	WP_000832235	(*fimH*) FimH protein precursor [Type 1 fimbriae (VF0221) - Adherence (VFC0001)] [*Escherichia coli* CFT073]
5	97558	98061	-	*fimG*	1-504/504	100.00	97.62	WP_000872015	(*fimG*) FimG protein precursor [Type 1 fimbriae (VF0221) - Adherence (VFC0001)] [*Escherichia coli* CFT073]
5	98074	98604	-	*fimF*	1-531/531	100.00	96.42	WP_001244821	(*fimF*) FimF protein precursor [Type 1 fimbriae (VF0221) - Adherence (VFC0001)] [*Escherichia coli* CFT073]
5	98614	101250	-	*fimD*	1-2637/2637	100.00	98.67	WP_000120946	(*fimD*) Outer membrane usher protein fimD precursor [Type 1 fimbriae (VF0221) - Adherence (VFC0001)] [*Escherichia coli* CFT073]

5	101317	102042	-	*fimC*	1-726/726	100.00	97.93	WP_000066579	(*fimC*) Chaperone protein fimC precursor [Type 1 fimbriae (VF0221) - Adherence (VFC0001)] [*Escherichia coli* CFT073]
5	102079	102618	-	*fimI*	1-540/540	100.00	99.07	WP_000824100	(*fimI*) Fimbrin-like protein fimI precursor [Type 1 fimbriae (VF0221) - Adherence (VFC0001)] [*Escherichia coli* CFT073]
5	102683	103231	-	*fimA*	1-549/549	100.00	92.17	WP_000695571	(*fimA*) Type-1 fimbrial protein A chain precursor [Type 1 fimbriae (VF0221) - Adherence (VFC0001)] [*Escherichia coli* CFT073]
5	103712	104308	-	*fimE*	1-597/597	100.00	98.83	WP_000044711	(*fimE*) Type 1 fimbriae Regulatory protein fimE [Type 1 fimbriae (VF0221) - Adherence (VFC0001)] [*Escherichia coli* CFT073]
5	104786	105388	-	*fimB*	1-603/603	100.00	97.84	WP_000790574	(*fimB*) Type 1 fimbriae Regulatory protein fimB [Type 1 fimbriae (VF0221) - Adherence (VFC0001)] [*Escherichia coli* CFT073]
5	272840	276498	-	*tsh*	182-3840/3840	95.26	95.57	WP_001528154	(*tsh*) temperature-sensitive protein Tsh [Tsh (VF0233) - Effector delivery system (VFC0086)] [*Escherichia coli* O25b:H4-ST131]
7	5487	6907	-	*espX1*	1-1422/1422	99.93	95.08	WP_000129338	(*espX1*) Type III secretion system effector EspX1 [TTSS secreted effectors (VF1110) - Effector delivery system (VFC0086)] [*Escherichia coli* O157:H7 str. EDL933]

Table S2. Antimicrobial resistance determinant characterization from whole genome sequencing data of U147 *E. coli* isolate.

CONTIG	START	END	STRAND	GENE	COVERAGE	% COVERAGE	% IDENTITY	ACCESSION	PRODUCT	RESISTANCE
21	19913	21022	-	*formA*_1	1-1110/1110	100.00	80.81	X73835	*formA*	Formaldehyde
26	30922	31713	+	*sul3*_2	1-792/792	100.00	100.00	AJ459418	*sul3*	Sulfamethoxazole
26	31971	32831	-	bla_{TEM-1B}_1	1-861/861	100.00	100.00	AY458016	bla_{TEM-1B}	Amoxicillin; Ampicillin; Cephalothin; Piperacillin; Ticarcillin
26	34720	35541	-	*lnu(F)*_1	1-822/822	100.00	100.00	EU118119	*lnu(F)*	Lincomycin
26	35675	36640	-	*ant(3")-Ia*_1	1-970/972	99.38	93.20	X02340	*ant(3")-Ia*	Streptomycin
26	39259	40119	-	*aac(3)-IId*_1	1-861/861	100.00	99.88	EU022314	*aac(3)-IId*	Apramycin; Dibekacin; Gentamicin; Netilmicin; Sisomicin; Tobramycin
32	25061	26686	+	*mcr-1.1*_1	1-1626/1626	100.00	100.00	KP347127	*mcr-1.1*	Colistin
36	1860	2675	+	*sul2*_2	1-816/816	100.00	100.00	AY034138	*sul2*	Sulfamethoxazole
36	5179	6392	-	*floR*_2	1-1214/1215	99.92	98.11	AF118107	*floR*	Chloramphenicol; Florfenicol
36	14968	15465	+	*dfrA12*_8	1-498/498	100.00	100.00	AM040708	*dfrA12*	Trimethoprim

36	15863	16664	+	*aadA2*_1	18-819/819	97.92	99.88	NC_010870	*aadA2*	Spectinomycin; Streptomycin
36	16926	18185	+	*cmlA1*_1	1-1260/1260	100.00	99.92	M64556	*cmlA1*	Chloramphenicol
36	18268	19069	+	*ant(3")-Ia*_1	171-972/972	82.51	99.75	X02340	*ant(3")-Ia*	Streptomycin
36	20997	22916	-	*tet(M)*_8	1-1920/1920	100.00	96.15	X04388	*tet(M)*	Doxycycline; Minocycline; Tetracycline
40	1624	2870	-	*tet(A)*_6	1-1247/1275	97.80	99.92	AF534183	*tet(A)*	Doxycycline; Tetracycline
53	138	953	-	*aph(3')-Ia*_1	1-816/816	100.00	99.88	V00359	*aph(3')-Ia*	Kanamycin

Table S3. Characterization of the IncX4 plasmid, that harbouring *mcr-1* gene, from U147 *E. coli* strain.

**Contig**	32
**Sequence_length(bp)**	33304
**GC_content(%)**	41.850828729281766
**Rep_type(s)**	IncX4
**Rep_type_accession(s)**	000224__NC_022105_00002
**Relaxase_type(s)**	MOBP
**Relaxase_type_accession(s)**	NC_013718_00057
**MPF_type**	MPF_T
**MPF_type_accession(s)**	NC_021079_00038,NC_013718_00045,NC_013718_00044,NC_013718_00041
**Predicted_mobility**	conjugative
**Mash_nearest_neighbor**	KX894453
**Mash_neighbor_distance**	0.000119496
**Mash_neighbor_identification**	*Escherichia coli*
**Primary_cluster_id**	AA619
**Secondary_cluster_id**	AI859
**Predicted_host_range_overall_rank**	family
**Predicted_host_range_overall_name**	Enterobacteriaceae

**Observed_host_range_ncbi_rank**	family
**Observed_host_range_ncbi_name**	Enterobacteriaceae
**Reported_host_range_lit_rank**	family
**Reported_host_range_lit_name**	Enterobacteriaceae
**Associated_pmid(s)**	26842776; 21911569; 28336940
